# Biomaterials in Maxillofacial Surgery: Membranes and Grafts

**Published:** 2011-06

**Authors:** Luigi F. Rodella, Gaia Favero, Mauro Labanca

**Affiliations:** 1 *Department of Biomedical Sciences and Biotechnology, Section of Human Anatomy, University of Brescia, Viale Europa 11, 25123 Brescia, Italy;*; 2 *Department of Dentistry, University “Vita e Salute”, San Raffaele, Milan, Italy*

**Keywords:** guided bone regeneration (GBR), reabsorbable membranes, bone grafts

## Abstract

Today, significant differences in the use of biomaterials (membranes and grafts) of animal or synthetic origin have yet to be reported. Nevertheless, some evidences suggest that synthetic materials have a lower risk of disease transmission. This review aims to assess the available informations on regenerative bone technique using reasorbable membranes and bone grafts. In particular, biocompatibility, immunological response, tissue reaction, reabsorption time and histological features of materials daily use in dentistry and in maxillofacial surgery were emphasized.

## GUIDED BONE REGENERATION

Guided bone regeneration (GBR) is a surgical method used to increase alveolar bone in patients with bone atrophy, before endosseous implant placement (1-3); this technique needs reasorbable membranes and bone grafts. The membranes prevent the invasion of surrounding soft tissue and permit to osteogenic cells to repopulate bone defects; bone grafts support the membranes and lead osteoblast growth (4-7).

Nowadays, there are several types of barrier membranes and graft materials used in GBR and each has both advantages and disadvantages (8, 9).

Regenerative bone materials, can be classified into four types according to their mechanism: *osteogenic materials*, which can directly stimulate bone cells to synthesize bone tissue; *osteoinductive materials*, which induce differentiation of mesenchymal cells into osteoblasts, improving bone formation in orthotopic and heterotopic sites; *osteoconductive materials*, which facilitate cell proliferation, migration and new bone apposition; *osteopromotive materials*, which act as a scaffold in which bone cells can grow (10-12).

Biomaterials, both membranes and grafts, can be also classified in relation to the following criteria: biocompatibility (immunological response), histological features and ability to maintain biological space.

Since 1982, when GBR technique was first introduced, the expanded polytetrafluoroethylene (e-PTFE) membrane has been considered the gold standard for barrier function materials (13). Indeed, this non reabsorbable material has all the features for GBR technique, such as biocompatibility, covering the defect and coagulum stabilization (14); nevertheless, e-PTFE membranes have also certain limits, such as the need of a second surgical operation to remove them and the possibility of bacteria infection (15-18).

Seibert and Nymann (1990) used e-PTFE non reabsorbable membranes to increase the alveolar crest; after 55-90 days, the bone completely filled up the defect (19). Recently, Urban and colleagues (2009) used e-PTFE membranes associated with autogenous grafts for implant insertions and shown that implant placed were osseointegrated and therefore the vertical GBR technique is both safe and predictable (13). Furthermore, a study by Ross-Jansaker (2007) has shown that perimplantar deficits should also be treated without membranes (20).

Recently, many types of membranes with a a lower risk of infections and/or contaminations that do not need a second surgical treatment have been produced and tested.

This review aims to analyse both membranes and bone grafts available to evaluate their advantages and disantvantages and to compare these biomaterials using fundamental parameters, such as biocompatibility, immunological response, tissue reaction, time of reabsorption and histological features.

## GBR FEATURES

### Biocompatibility

Biocompatibility is a fundamental condition for the use of biomaterials (9). Hartwing (1972) defined that a biomaterial is compatible with the surrounding tissue if the interface between vital tissue and material is similar to natural zones, without induce inflammation or immunological response (21).

### Time of reabsorption

Reabsorbable materials remain *in situ* until new bone tissue regenerates, so they could increase implant osseointegration. Different animal models affect the time of biomaterial reabsorption.

### Biological space

There are different types of membranes commercially available today: membranes that create a correct biological space (semi-hard synthetic membranes), membranes with restricted ability to create it (synthetic membranes) or membranes that do not maintain the biological space (collagen membranes).

However, reabsorbable membrane increases bone regeneration especially when associated with bone graft (13, 22).

## REABSORBABLE MEMBRANES

Several studies on reasorbable membranes have been conducted to evaluate the conditions associate with different experimental and human models. In particular, Gottlow (1984) shown that a biological space induced correct bone regeneration, while without this space the membrane collapsed and so compromised bone regeneration (23). The isolated space created could be invaded by osteogenetic cells from the surrounding bone and therefore induced bone regeneration. This principle has been confirmed by numerous authors. Dahlin (1989) studied the recovery of alveolar defects with or without the use of membranes and shown those 6 weeks after treatment with membrane the defects had completely covered by new tissue, while defects without membranes were covered by fibrous tissue, even if it was reduced (24). Similar results were obtained by Kostopoulos and Karring (1994) using reabsorbable membranes to repair defects of the inferior side of the mandibular branch (25).

Moreover, studies on bone deficit underlined the importance of membrane porosity to increase osseointegration and tissue vascularization near the implant (26, 27).

One of the first requirements for membrane biocompatibility is permeability to body fluids. When membranes are applied to regenerate tissue, additional features, such as cell-occlusive properties and biocompatibility, become important. These properties contribuite to membrane stabilization, integration into soft tissue and degradation in the case of reabsorbable membranes (5). The reabsorbable membranes are classified as homologous membranes (human *dura mater*), heterologous animal membranes or synthetic membranes.

### Human dura mater membranes

Dori *et al*. (2008) shown that *dura mater* membrane was completely reabsorbed. In spite of treatment with γ rays, infective diseases, such as AIDS and Creutzfeld-Jacob disease (CJD) can be transmitted with a risk between 1:10000 and 1:100000 (28, 29).

### Heterologous animal membranes

Collagen membranes are biomaterials derived mainly from bovine source and made of collagen types I and III (30). The reabsorption of these membranes is due to the action of collagenases that cleave the collagen in two molecules which are denaturized at 37°C and decomposed into oligopeptides and aminoacids by the gelatinase and proteinase (31).

The time of reabsorption can be modified by cross-linked treatment, the cross-linking with glutaraldehyde reduce the inflammatory response and prevent degradation of the membranes since 30 days, so these membranes are useful when the synthesis of new bone depends on the prolonged presence of a mechanical barrier (32). Miller (1996) used membranes cross-linked with acid-azide and an amminc solution to quicken reabsorption; nevertheless this modification induced an inflammatory response (33). Hyder (1992) observed that collagen membranes started degradation after 21 days, while after 35 days there were only a few areas of collagen residual (34). Moreover, in human, Van Swol (1993) shown that bovine derma collagen membranes degraded after 3 months (33). Recent studies have shown that the new collagen membranes are completely reabsorbed in 6 months (30).

### Synthetic membranes

Synthetic membranes are formed by polylactic acid and recently also with polyglycolic acid and citric acid esters, in order to decrease the rapidity of reabsorption and increase their malleability (9, 34). The reabsorption of synthetic membranes is through the Krebs cycle: glycolic copolymers are split up into lactic acid and pyruvate, which are directly induced in the citric acid cycle and so eliminated through the formation of carbon dioxide and water (35).

Hyder (1992) and Kodama (1989) noted that the inflammatory infiltrate induced by synthetic membranes was lower than heterologous animal membranes (34, 36). Robert and Frank (1994) showed that, changing the polymer concentration, the membranes hold out for about 4 months (37). Laurel (1994) underlined a time of reabsorption between 6 and 12 months, but the hydrolyses of the membrane caused little inflammation (38).

Many studies have shown that time of reabsorption for this kind of membranes is about 6 months (39-45); while for Miller (1996) the synthetic membranes are reabsorbed slowly and should cause infiammatory response compared to collagen membranes (33).

Different bioreabsorbable polymers and co-polymers are presently used in synthetic membranes and the poly-DTE-carbonate has shown promising features, such as low immunological reaction and high ability to induce bone regeneration (46). Polylactic acid-polyglycolic acid (PLLA-PGA) co-polymer provides a rigid scaffolding to secure the graft materials. The clinical application of these co-polymer membranes may be useful for periodontal reconstructive procedures, such as GBR (47).

## GRAFT MATERIALS

Grafts are fundamental for regenerating and repairing of bone tissue. Several types of filling biomaterials have been evaluated for bone regeneration and the choice of the biomaterial mostly depends on its features and application site (48). The grafts could be classified as autologous, homologous, heterologous and synthetic materials (49, 50).

### Autologous grafts

Since 1978, autologous material has been used for bone regeneration and presently it is considered the gold standard in bone grafts since it has osteogenetic, osteoconductive and osteoinductive features (51-53). Graft integration in bone defect needs correct vascularisation both through neo-synthetized vessels and anastomosis between the vessels of the receive site and of the bone graft.

Histomorphometric analysis shown 42% of neo-synthetized bone, 40% of medullary spaces and 18% of residual autologous bone (54). There is no immunological response to autologous grafts. Its main disadvantages are increased surgical time and patient morbidity (55, 56).

### Homologous grafts (Allografts)

Vital bone tissue is obtained from donors and it is stocked in bone banks (10). The use of homologous grafts is limited, due to the risk of infection, in particular the risk of contracting HIV is estimated to be 1:1.6 million, compared with 1:450000 in blood transfusions. Rigorous background checks must be made on the donor and his/her family (11). Homologous grafts, before their use, are tested and treated to prevent any risk of antigenicity or diseases transmission (9, 49, 57).

The bone should be lyophilized and demineralized (DFDB - Demineralized Freeze-Dried Bone) or only lyophilized (FDB - Freeze-Dried Bone). In particular, Yukna and Vastardis (2005) compared the histological results of bone defects filled with FBD or with DFDB and noted more regenerate bone tissue with FBD (58). Moreover, Dahlin (2010) shown that the reconstruction of atrophic maxillae with DFDB in combination with GBR technique can be performed with equal treatment outcomes and a significant reduced cost compared with autologous bone from iliac crest (59).

Contradictory opinion about the properties of allografts are present in literature. Whittaker (1989) and Kubler (1993) asserted that allografts have both osteoinductive and osteoconductive properties (60, 61), while Wetzel (1995), Becker (1995), Frost (1982) disagreed with this hypothesis and asserted that have only osteoinductive ability (62, 64). The histomorphometric analysis has shown 29% of neo-synthetized bone, 37% of medullary spaces, while 34% of DFDB residual particles (54); moreover, the replacement of homologous bone is slow (12) and it causes the formation of connective areas and where graft integration is reduced there is a visible inflammatory infiltrate.

### Heterologous grafts (Xenografts)

Heterologous materials are obtained from bones of different animal species; bovine bone being the most common source (65). Xenografts have different properties depending on their origin, constitution and processing (10).

**Bovine grafts.** Bovine bone xenografts have been used in several types of bone defects with satisfactory results (2, 10). These biomaterials are made of apatite crystals in a reticular form, with an inside surface of about 70 m^2^/g which induces coagulum synthesis and stability (67).

Many authors have confirmed their osteoconductive properties (68-71). Nevertheless, there is always a risk of trasmission of CJD or Bovine Spongiform Encephalopathy (72) according to the Food and Drug Administration (FDA).

Histomorphometic analysis has shown 39% of new bone, 34% of medullary spaces and 27% of residual bovine material (54). This biomaterial has low reabsorption: after many years the material is still between 20-40%. Histological analysis performed by Hallman and Lundgren (2001) shown that the percentage of grafts after 6 months is equal to that visible after 3 years of placement (73).

**Bovine collagen grafts.** Collagen contributes to mineral deposition, vascular ingrowth and growth factor binding, so provides a favorable environment for bone regeneration. Since 1990, the FDA has demonstrated that this biomaterial could induce allergic responses; in fact, 3% of the population is allergic to the collagen and so has a predisposition to develop diseases such as polymyositis and dermatomyositis (71).

### Alloplastic grafts

Alloplastic grafts are synthetic bone substitutes that are available in different sizes, forms and textures (10, 11, 48). Bauer and Mischler (2000) noted that this type of bone graft can induce stable bonds with neo-synthetized bone (77). The structural characteristics of the alloplastic grafts are similar to bone tissue (75). In particular, Sasaoka (1989) reported that bioactive ceramics, a type of synthetic graft, bind bone naturally, due to their similarity with mineral bone tissue (76). Stavropoulos and colleagues (2004) compared the performance of synthetic reabsorbable materials (PGA-TMC, glycolide 67% plus trimethylene carbonate 33%) with animal origin reabsorbable membranes (collagen membranes) and demonstrated that the quality of new bone was significantly higher in the group treated with PGA-TMC compared with the group using only collagen membranes (77).

**Hydroxyapatite allografts.** Hydroxyapatite is a natural component of hard tissue (65% in bone tissue, 98% in enamel). Synthetic hydroxyapatite is available in different forms: porous, non- porous, ceramic and non-ceramic.

This material has been used in GBR techniques to coat implants, due to its osseointegrative capabilities (11, 78, 79).

Hydroxyapatite is bioinert and biocompatible, but it does not induce significant bone regeneration. Histomorphometric analysis resulted in a percentage of 41% of neo-synthetized bone, 30% of medullary spaces and 31% of residual hydroxyapatite graft (54), so it is poorly reabsorbed.

**Tricalcic phosphate grafts.** Tricalcic phosphate grafts (Ca_3_(PO_4_)_2_) is treated with naphthalene and then is compacted at 1100-1300°C to obtain a diameter porosity of 100-300 μm. The studies of Koyama (2007) had shown an increase of bone regeneration after 12 weeks from surgery placement (4).

Moreover, during reabsorption, it provides ion calcium and magnesium to bone tissue and so creates a correct ionic environment, which induces alkaline phosphatase activation, fundamental for bone synthesis (80, 81).

**Bioglass grafts.** Synthetic glass ceramics are made of silicon dioxide (45%), sodium oxide (24.5%) and phosphorus pentoxide (11, 82). The bioglass is used mainly in maxillary sinus lifts and is characterized by particles with a diameter of 300-335 μm. Bioglass has osteoconductive properties and their solubility is directly dependent on sodium oxide (11).

Histomorphometric analysis has given a percentage of 40% of new bone, 43% of medullary spaces and 17% of bioglass particles surrounded by neo-synthetized bone (54, 82).

**Coralline hydroxyapatite grafts.** Coralline hydroxyapatite is composed of calcium carbonate (87-98%), strontium, fluoride, magnesium, sodium and potasium (2-13%) (11, 83). It has a porous structure (over 45%) and pores have a diameter of 150-500 μm.

Guilemin (1987) underlined that these grafts are higly biocompatible (84). The coralline hydroxyapatite also has osteoconductive properties (85) and the reabsorption of the coralline skeleton is due to the action of the carbonic anhydrase of osteoblasts (86).

Histomorphometric analysis shown 42% of neo-synthesized bone, 40% of medullary spaces and 18% of residue biocoral (54).

**Polylactic acid and polyglycolic acid.** The union of polymeric lactic acid and polyglycolic acid increases graft compatibility and degradability (9, 82). Cauwels and Martens (2004) found that this graft not induced inflammatory processes, confirming the biocompatibility of this material (88). The insertion of polylattic and polyglicolic acid biopolymers induces a correct bone regeneration (89-91). Histological analyses shown that the graft was almost completely reabsorbed; in particular, the histomorphometric analysis shown 43% of mineral bone, 56% of medullary spaces and only 1% of residual graft (54). The degradation and reabsorbition of this material is slow and progressive, inducing a correct bone regeneration. Reabsorption is about 4-8 months due to the low density of the product.

New membranes made of polylactic acid (PLA), aminopropyltriethoxysilane (APTES) and carbonate of calcium show greater ability to induce bone cells proliferation compared to non-hybrid membranes (92); nevertheless, other clinic studies are necessary to confirm these results.

The data about the graft material’s histomorphometric analysis are summarized in Table 1.

**Table 1:** Histomorphometric analysis of different graf materials (summarized from Piattelli, 2003)

Graft material	Feature	neo-synthetized bone (%)	medullary space (%)	residual graft (%)

Autologous graft	Osteogenic, osteoconductive, osteoinductive properties	42	40	18
Homologous graft (Allograft)	Osteoconductive and osteoinductive properties	29	37	34
Heterologous graft (Xenograft)	Osteoconductive property			
Hydroxyapatite graft		41	30	31
Bioglasses		40	43	17
Biocoral		42	40	18
Polylactic acid and polyglycolic acid		43	56	1

## CONCLUSION

In GBR technique, many graft materials can be chosen and many relative factors have to be considered, such as bone defect site, surgical objective, patient examination and knowledge of graft materials (2, 11). The graft materials have not to induce inflammation responses and they have to be osteoconductive to maintain trophism under the membrane and rapid reabsorption (8, 9).

From a professional point of view, the results and performances obtained by different biomaterials (membranes and grafts) do not underline clearly differences within bone regeneration induced by heterologous materials from animal origin or synthetic materials (22, 42, 93, 94).

There are no significant differences, reported in literature, in the use of animal heterologous grafts or synthetic alloplastic grafts. Nevertheless, it is our opinion, that a correct choice is fundamental to minimize the possibility of disease transmission and development; in particular, synthetic biomaterials are better compared to heterologous animal biomaterials, which have a higher risk of inflammatory reactions and disease trasmission.
